# ‘Standing together – at a distance’: Documenting changes in mental-health indicators in Denmark during the COVID-19 pandemic

**DOI:** 10.1177/1403494820956445

**Published:** 2020-09-10

**Authors:** Amy Clotworthy, Agnete Skovlund Dissing, Tri-Long Nguyen, Andreas Kryger Jensen, Thea Otte Andersen, Josephine Funck Bilsteen, Leonie K. Elsenburg, Amélie Keller, Sasmita Kusumastuti, Jimmi Mathisen, Amar Mehta, Angela Pinot de Moira, Morten Hulvej Rod, Morten Skovdal, Katrine Strandberg-Larsen, Ingrid Willaing Tapager, Tibor V. Varga, Johan Lerbech Vinther, Tianwei Xu, Klaus Hoeyer, Naja Hulvej Rod

**Affiliations:** 1 Department of Public Health, University of Copenhagen Faculty of Health and Medical Sciences, Denmark; 2 Steno Diabetes Center Copenhagen, Denmark; 3 Statistics Denmark, Copenhagen, Denmark

**Keywords:** Mental health, COVID-19, quality of life, social isolation, worries, public health, Denmark, citizen science

## Abstract

*Aims:* There is a need to document the mental-health effects of the COVID-19 pandemic and its associated societal lockdowns. We initiated a large mixed-methods data collection, focusing on crisis-specific worries and mental-health indicators during the lockdown in Denmark. *Methods:* The study incorporated five data sources, including quantitative surveys and qualitative interviews. The surveys included a time series of cross-sectional online questionnaires starting on 20 March 2020, in which 300 (3×100) Danish residents were drawn every three days from three population groups: the general population (*N*=1046), families with children (*N*=1032) and older people (*N*=1059). These data were analysed by trend analysis. Semi-structured interviews were conducted with 32 people aged 24–83 throughout Denmark to provide context to the survey results and to gain insight into people’s experiences of the lockdown. *Results:* Absolute level of worries, quality of life and social isolation were relatively stable across all population groups during the lockdown, although there was a slight deterioration in older people’s overall mental health. Many respondents were worried about their loved ones’ health (74–76%) and the potential long-term economic consequences of the pandemic (61–66%). The qualitative interviews documented significant variation in people’s experiences, suggesting that the lockdown’s effect on everyday life had not been altogether negative. ***Conclusions:* People in Denmark seem to have managed the lockdown without alarming changes in their mental health. However, it is important to continue investigating the effects of the pandemic and various public-health measures on mental health over time and across national contexts.**

## Introduction

The novel coronavirus severe acute respiratory syndrome (SARS) coronavirus 2, or coronavirus disease 2019 (COVID-19), has caused a global pandemic [1]. To reduce its transmission, international health authorities have promoted specific preventative measures, including self-isolation, increased hand hygiene and physical distancing [2]. Many countries have begun to document the effects of the pandemic and its related societal lockdowns/quarantines on mental health [3–11]. In China, quarantining seems to have caused an increase in fear, stress and anger, especially amongst survivors of previous outbreaks (i.e. SARS and Ebola), frontline health-care workers and people younger than 35 years of age [12,13].

On 27 February 2020, the first COVID-19 case was documented in Denmark; on 11 March, the government and health authorities announced a lockdown of educational activities and many jobs. Legal sanctions were instituted against public gatherings of more than 10 people, and citizens were strongly encouraged to stay home and to maintain a distance of two metres from others [14]. The official public-health recommendations emphasised protecting vulnerable people with an increased risk for severe outcomes: that is, people aged 65+ and people with compromised immune systems and/or chronic illnesses (e.g. cardio-metabolic or lung diseases, etc.) [15]. The government also negotiated relief packages with labour unions and employer organisations to support the economy and reduce financial anxiety.

The public-health recommendations and societal lockdown affected everyday life in Denmark as numerous employees suddenly worked from home, parents home-schooled their children and many businesses were temporarily closed. Because both parents in most families work full time, the lockdown of day-care institutions and schools was expected to put considerable strain on families with young children. Although research has begun investigating Danes’ reactions to the so-called corona crisis [16–18], there is a lack of knowledge about how the chosen public-health measures impacted mental health. Specifically, how has the pandemic together with the particular Danish combination of relative economic security, a lockdown of certain societal functions and only partial restrictions on movement affected mental health?

To address this, we established an interdisciplinary research project ‘Standing together – at a distance’, and initiated a series of timed online surveys and qualitative interviews to document the immediate effects of the Danish lockdown on mental health amongst different population groups. This collection aimed to investigate how the pandemic and its related public-health measures affected people’s worries, quality of life, social isolation, relationships and everyday behaviour.

This paper provides an overview of our study design to ensure transparency and promote international comparisons. We also present some of our initial findings, focusing on crisis-specific worries and changes in mental-health indicators over time within three groups: the general population, families with children living at home and older people. We expected the lockdown to affect the latter two groups significantly. The analysis presented here is not a full reporting of the data. Rather, we aim to illustrate how these data can be used and understood in order to invite collaborations with both Danish and international researchers. This research may elucidate long-term effects of the societal lockdown in Denmark, which may thereby inform governments and health authorities in how to manage both the current and future pandemics more effectively.

## Methods

### Project overview

**Figure 1.**
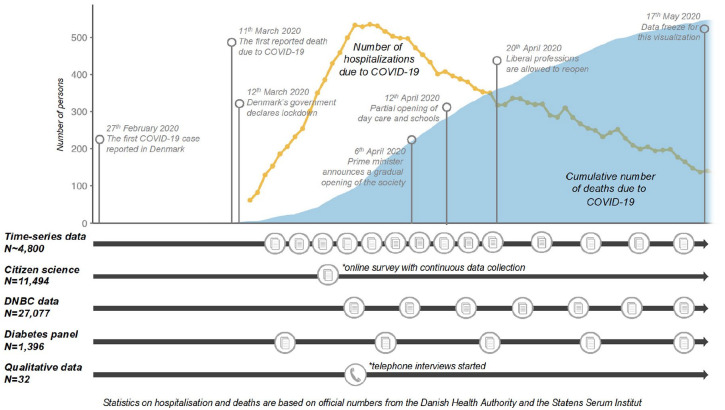
Timeline showing seminal events during the lockdown in Denmark in relation to how and when the project collected data between 27 February and 17 May 2020. The hospitalisation curve represents the total number of patients hospitalised at any given time, while the death curve represents the cumulative number of deaths due to COVID-19.

The ‘Standing together’ project represents an interdisciplinary collaboration between researchers at the University of Copenhagen’s Department of Public Health, the Danish National Birth Cohort (DNBC), Steno Diabetes Center Copenhagen and the Danish Diabetes Association. The project is based on two interrelated components: quantitative surveys and qualitative interviews. Alongside the real-time data collection, we documented the political initiatives that were implemented to create timelines of the economic relief packages and laws passed. Figure 1 shows the seminal events and announcements from the Danish government in relation to when and how we collected data from late February until mid-May 2020.

### Copenhagen Corona-Related Mental Health Questionnaire

We designed the Copenhagen Corona-Related Mental Health (CCMH) questionnaire to focus on mental-health indicators, worries and behaviours related to the COVID-19 crisis. The questionnaire includes: sociodemographic measures (i.e. age, sex, education, postal code and occupation); COVID-19 symptoms, diagnosis and hospitalisation; chronic physical and mental disorders; perceived social isolation; loneliness (UCLA short three-item T-ILS version) [19]; common mental disorders [20]; crisis-related mental-health indicators (i.e. anxiety, loneliness, hopelessness, depression and physical stress symptoms); quality of life; quality of sleep; COVID-19-related precautions and worries; and sources of information. The CCMH questionnaire was used in the quantitative data sources presented below.

### Data sources

#### Time-series data

In collaboration with the consumer research company Epinion [21], we initiated a time series of cross-sectional online surveys with 300 (3×100) Danish residents drawn every three days from three population groups: people aged 65 and above, families with children living at home and the general population aged 18–87. Starting on 20 March, data were collected every three days; from 16 April until 25 June, they were collected once a week. We collected data from approximately 2100 people within each population group (*N*=6300). Additional rounds of data collection are planned to begin in September 2020.

#### Citizen-science sample

Our website (https://coronaminds.ku.dk/english/) was established as an interactive platform to engage the public. Here, we regularly publish results from incoming data, and via a dedicated link launched 26 March, we recruit additional respondents to answer the CCMH questionnaire. This recruitment is a collaboration with the Danish newspaper *Politiken*, which published the initial results along with a link to our website. This data collection is ongoing; as of 17 May, 11,494 people had participated in the questionnaire via our website.

#### Birth-cohort data

Between 30 March and 2 April, an online survey was initiated in the DNBC [22]. Participants with a valid email address or telephone number were invited; that is, 53,323 adolescents born into the cohort (now aged 16–24) and 53,968 mothers who enrolled in 1996–2002 while they were pregnant. During the first data collection, data were available for 13,002 adolescents (response rate=24%) and 14,075 mothers (response rate=26%). Respondents who completed the first questionnaire within a week were invited to participate in weekly surveys until 14 May 2020. Another data collection is planned for September 2020.

#### Diabetes-panel data

Between 19 and 26 March, 2430 adults (aged >18) who are members of two user panels at Steno Diabetes Center Copenhagen and the Danish Diabetes Association received online questionnaires. The panels represent people being treated in primary- and/or secondary-care settings across Denmark who have been diagnosed with diabetes mellitus type 1 or 2, maturity onset diabetes of the young, gestational diabetes or other rarer types of diabetes. In total, 1366 individuals responded to the questionnaire (response rate=56%), and were invited to complete five repeated surveys until 14 May [23]. (For a comprehensive overview of response rates in the DNBC and diabetes-panel data, see Supplemental Material 1. Response rates are not available for the time-series data and the citizen-science sample collected by Epinion.)

#### Qualitative data

To contextualise the CCMH questionnaire results, semi-structured qualitative interviews [24] were initiated on 30 March. These were conducted via telephone or safe-link video call with people recruited from three survey populations: (a) the citizen-science sample, (b) the time-series sample and (c) the diabetes panel. With the time-series sample, we asked Epinion to select from three groups: people aged 70 and above, parents with school-aged children (to align with the questionnaire’s target groups) and people with chronic illnesses. We aimed for maximum variation regarding geographical area, employment status, different chronic illnesses and, within the family group, number of children. As of 17 May, we had conducted 32 interviews with people aged 24–83 throughout Denmark.

### Analyses of the initial survey results

Using the time-series data, we conducted descriptive analyses of crisis-specific worries and mental-health indicators based on data collected from 20 March to 16 April.

#### Measurements

**Figure 2.**
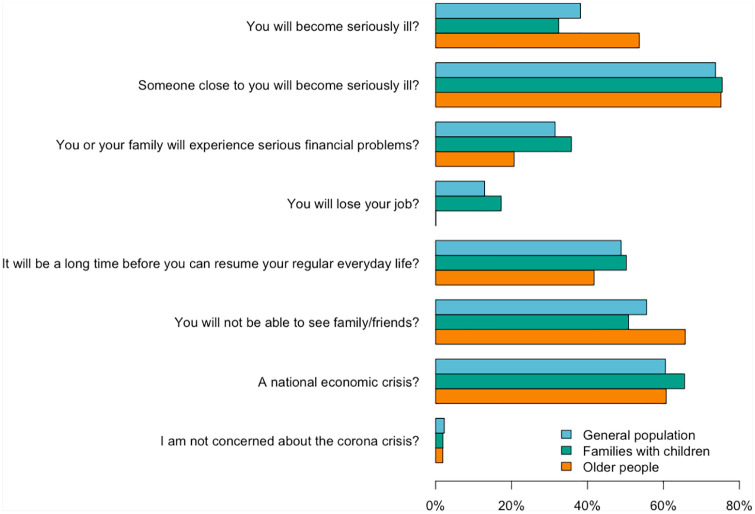
Crisis-specific worries within the general population (*N*=1046), families with children (*N*=1032) and older people (*N*=1059) based on data collected from 20 March to 16 April 2020.

Participants answered an item regarding crisis-specific worries: ‘What makes you worried about the corona crisis?’ Multiple response categories were allowed (see Figure 2). Participants also answered items regarding mental-health indicators, rated on a scale from 1 to 10: ‘How worried are you about the corona crisis?’, ‘How would you rate your quality of life right now?’ and ‘How socially isolated do you feel right now?’.

#### Statistical methods

We calculated proportions of affirmative responses regarding crisis-specific worries within the three population groups using data averaged over time. To increase representativeness, the general population was weighted on age, sex and geographical region using raked-weighting methodology [25] and data from the Danish population register ‘FOLK1A’ (see Supplemental Material 2 for the distribution of weighting variables). To investigate changes in mental-health indicators over time, we estimated means and 95% credible intervals [26] for each indicator and estimated the Trend Direction Index (TDI) [27]. The TDI estimates the probability that the population average was increasing or decreasing at a given time for each indicator. For example, a TDI of 80% at a specific time point means that the average indicator is increasing at that time, with a probability of 80% implying 20% probability for it to be decreasing. A TDI of 50% marks the change between it increasing or decreasing. This analysis allowed us to compare putative trends in the population’s mental health with specific events at different time points.

### Analysis of the qualitative interviews

We asked questions that specifically aligned with the CCMH questionnaire but enabled people to elaborate and describe their own experiences. These interviews aimed to complement the quantitative results and to enrich them with narrative insights into how people in Denmark experienced the pandemic and the public-health recommendations. Each interview was transcribed and thematically coded [28], and a pseudonym was assigned to protect each person’s identity [29]. We also removed any identifying features from their quotes. The quotations included here are our own translations from Danish to English.

## Results

### Sociodemographic characteristics and crisis-specific worries

Table I shows the sociodemographic characteristics of the three groups captured in the time-series data; Figure 2 shows crisis-specific worries. In all three groups, most people were worried about someone close to them becoming seriously ill (74–76%). The majority were also worried about a potential national economic crisis (61–66%). Compared to the general population and families with children, older people were worried about becoming seriously ill themselves (54% vs. 32–38%) and not being able to see their family and friends (66% vs. 51–56%). More families with children than older people were worried about experiencing serious financial problems (36% vs. 21%) and not being able to resume everyday life (50% vs. 42%). In each population group, approximately 2% were not worried about the COVID-19 crisis. See Supplemental Material 3 for descriptive changes in the crisis-specific worries over time.

**Table I.** Sociodemographic characteristics within the population groups (time-series data).

	General population (*N*=1046)	Families with children (*N*=1032)	Older people (*N*=1059)
Age (years), mean (lowest age – highest age)	49 (18–87)	41 (18–77)	72 (65–89)
Women, *n* (%)	530 (51)	510 (49)	575 (54)
Short-cycle higher education, *n* (%)	105 (10)	103 (10)	114 (11)
Medium-cycle higher education, *n* (%)	317 (30)	344 (33)	429 (41)
Long-cycle higher education, *n* (%)	192 (18)	221 (21)	142 (13)
Other education,^a^ *n* (%)	432 (41)	364 (35)	374 (35)
Living alone, *n* (%)	256 (24)	0 (0)	354 (33)

a Primary school, high school, vocational education, other education.

### Changes in mental-health indicators over time

**Figure 3.**
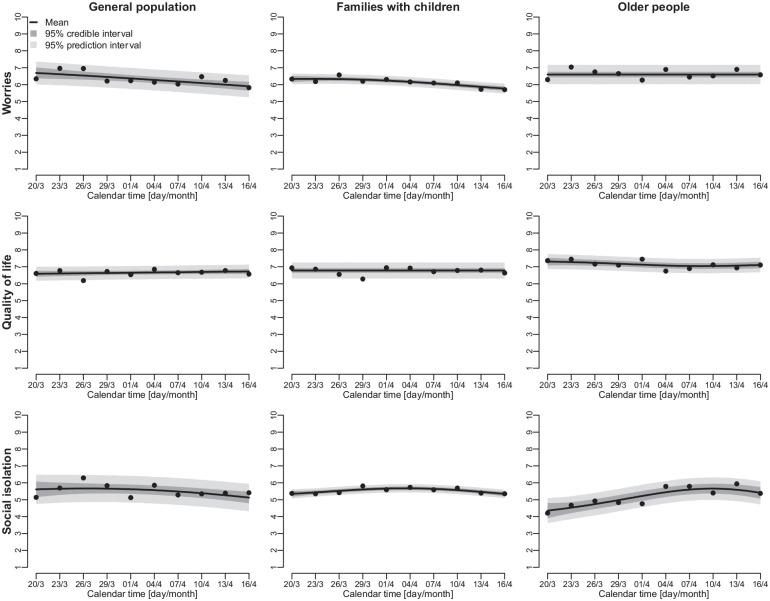
Changes in mental-health indicators from 20 March to 16 April 2020; means, 95% credible intervals (dark grey) and 95% prediction intervals (light grey).

**Figure 4.**
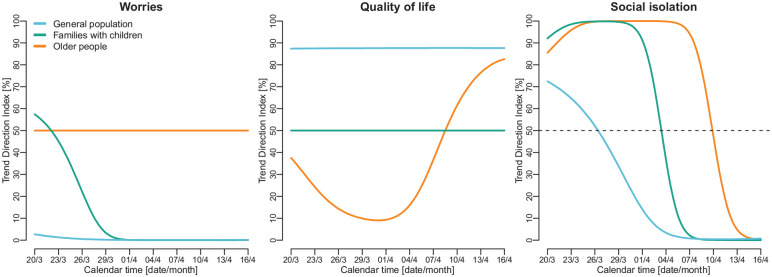
The Trend Direction Index (TDI) for the period 20 March to 16 April 2020, indicating the probability that the population average of each mental-health indicator was increasing or decreasing at specific time points.

Absolute levels of worries, quality of life and social isolation remained relatively stable during the observation period, with variations of only ±1–2 points on a 10-point scale (Figure 3). However, the trend analysis revealed some interesting underlying trends within the three population groups (Figure 4).

For the general population, there was a decreasing trend for worries over the observation period (TDI=97–100%), while there was an increasing trend for quality of life (TDI=87%). Feelings of social isolation increased slightly within the general population (TDI=72%) at the start of the observation period, but this trend reversed around 26 March. From 1 April, the TDI indicated with >86% probability that feelings of social isolation were decreasing within the general population.

A different picture emerged for older people. Their level of social isolation increased throughout most of the observation period (TDI=86–95% from 20 March to 7 April) but began to decrease around 13 April (TDI=95%). Similarly, older people’s quality of life decreased during the first weeks of the lockdown (TDI=9–37%) but then increased from around 9 April (TDI=61–86%).

Amongst families with children, there were initially increasing trends for worries and social isolation from approximately 29 March, followed by decreasing trends in worries and social isolation from 4 April. In contrast, this group’s quality of life remained relatively constant throughout the period (TDI=50%).

### Qualitative interviews

Most of our interviews were with women (*N*=22), and four people identified as non-Danish. These interviews provided insight into how people in Denmark experienced the lockdown as it developed (see the corresponding timeline in Figure 1). Although some found it ‘boring’ and others compared it to being in ‘prison’, most supported this measure and considered it manageable. Lise (age 81) said, ‘I really think I’ve done well. I thought it would have been worse’, adding, ‘Of course, I miss contact [with] my children and grandchildren’ (interview; 16 April). Some people highlighted positive aspects, such as being able to slow down and contemplate what matters in life. Annette (age 48), who was on sick leave for anxiety when the lockdown started, found it almost helpful: ‘It’s like it was okay to pull the plug when the rest of Denmark reduced its speed’ (3 April).

Many older people have been isolated to protect them from infection, but this caused their relatives to express concern. Annette said, ‘I’m nervous about my mother-in-law . . . she’s 86 and lives alone. She probably won’t get sick . . . But she’s really alone in this situation’ (3 April). Karen (age 64) said, ‘My mother lives . . . in a care home, and she’s locked up there. You can’t come in to visit her, and she can’t get out. And you don’t [know] what’s happening because she can’t talk on the phone’ (30 March). Families with children also mentioned the lockdown’s isolating effects. Anja (age 39), mother of two children (ages 7 and 2), said, ‘Our everyday life has changed significantly because we’re at home instead of being out in the world . . . We haven’t seen anyone for 20 days now . . . I think that’s difficult . . . The children miss getting input from someone other than us. And, personally, I also miss having contact with other people’ (31 March).

At the beginning of the crisis, many people indicated that they were worried about a close relative becoming seriously ill. However, some people with chronic illnesses, who are considered a ‘high-risk’ group, described their relatives as ‘hysterical’ or ‘overprotective’. This suggests that people with chronic illnesses, who may have spent years successfully managing risk, may feel disempowered. Interestingly, some members of the ‘high-risk’ groups were less concerned about their own health, worrying instead about the country’s socio-economic stability.

## Discussion

This paper presents a mixed-methods study focusing on changes in mental-health indicators during the COVID-19-related lockdown in Denmark. In the quantitative surveys, absolute level of worries, quality of life and social isolation were relatively stable during the first six weeks, with the TDI analysis revealing specific underlying trends within the three population groups. Our initial results indicate that many people – even those in ‘high-risk’ groups – were more worried about others becoming seriously ill than themselves. In the qualitative interviews, people with chronic illnesses described their relatives as overly protective.

In both the surveys and interviews, people expressed concern about a possible national economic crisis. While most mental-health changes related to the pandemic will hopefully resolve as Danish society reopens and the virus’s spread is controlled, there may be long-term consequences for both the country and individuals. Despite the relief packages implemented to support the economy and to reduce financial anxiety, unemployment and economic stress are well-known risk factors for mental-health problems throughout the life course [30].

In our study, time variations in mental-health indicators were found to be small, which aligns with other studies measuring mental-health indicators over time during the Danish COVID-19 lockdown [17,18]. Interestingly, the general population and particularly families with children seemed to experience a slight decrease in their levels of social isolation following the announcement on 6 April of a gradual reopening of schools and day-care institutions. Moreover, older people had generally higher levels of worry throughout the observation period, experiencing the greatest decrease in quality of life and the greatest increase in social isolation, although absolute differences were small. One explanation for these differences could be that compared to other groups, older people may have to endure a longer period of or more restrictions; some might also not use digital technologies, which can hinder online social interaction. It is worth noting that older adults who participate in online questionnaires are likely to be high functioning. Thus, the results may not be representative for all older people, particularly those living in long-term care facilities.

Another key finding is the significant variations in people’s individual experiences of the lockdown, which was reflected in both the surveys and the interviews. At different time points, some felt confined, while others appreciated slowing down. This suggests that rather than focusing solely on means and averages, future studies should aim to integrate qualitative interviews and quantitative data to understand such differing reactions better. Our project has benefitted from combining these components; that is, the interviews contextualised certain changes in mental-health indicators during the lockdown. A particular strength of our study is that by triangulation across various study designs and methods, we can further investigate such variations in future analyses.

The collected data provide rich possibilities to analyse mental-health changes within certain vulnerable groups, such as people with existing chronic illnesses and/or psychiatric disorders. It would also be valuable to investigate whether worries, loneliness and social isolation are more severe within particular groups that other research has identified as having a higher risk for infection or financial hardship, and thus obtain background parameters for worries, loneliness and isolation within particular groups. Moreover, it would be productive to compare varying levels of financial stress in different countries, particularly welfare societies, and to engage in other international comparisons.

It should be noted that although we weighted data on key variables (i.e. age, sex and region), it was not possible to weight on other important factors such as socio-economic position. Moreover, the mental-health measures used are not validated measures. Rather, they should be viewed as overall indicators of mental health during the COVID-19 pandemic in Denmark.

## Conclusions

Our initial findings indicate that people living in Denmark have managed the COVID-19 pandemic and its associated societal lockdown without alarming changes to their immediate levels of worry, quality of life or social isolation. However, people expressed concern about their loved ones’ health and potential long-term socio-economic consequences of the lockdown. It is important to continue investigating the effects of the pandemic and various public-health measures on mental health in different countries. We hope our data can contribute to future analyses of mental-health developments over time and across national contexts.

## Supplemental Material

CEP956445_Supplemental_material – Supplemental material for ‘Standing together – at a distance’: Documenting changes in mental-health indicators in Denmark during the COVID-19 pandemic Click here for additional data file. Supplemental material, CEP956445_Supplemental_material for ‘Standing together – at a distance’: Documenting changes in mental-health indicators in Denmark during the COVID-19 pandemic by Amy Clotworthy, Agnete Skovlund Dissing, Tri-Long Nguyen, Andreas Kryger Jensen, Thea Otte Andersen, Josephine Funck Bilsteen, Leonie K. Elsenburg, Amélie Keller, Sasmita Kusumastuti, Jimmi Mathisen, Amar Mehta, Angela Pinot De Moira, Morten Hulvej Rod, Morten Skovdal, Katrine Strandberg-Larsen, Ingrid Willaing Tapager, Tibor V. Varga, Johan Lerbech Vinther, Tianwei Xu, Klaus Hoeyer and Naja Hulvej Rod in Scandinavian Journal of Public Health
