# Supplementary material for: ‘Standing together – at a distance’: Documenting changes in mental-health indicators in Denmark during the COVID-19 pandemic
Source: Scand J Public Health. 2020 Sep 10;49(1):79–87. doi: 10.1177/1403494820956445 (PMC7859573; doi:10.1177/1403494820956445)

Supplemental Material

SJPH manuscript SPUB-RI-2020-0220

'Standing together – at a distance': documenting changes in mental-health indicators in Denmark during the COVID-19 pandemic

Supplemental material 1

Supplemental material 1: Overview of the data collections and response rates in the Diabetes panel and the Danish National Birth Cohort (DNBC)

	Diabetes panel					Birth Cohort data (DNBC)				
Data collection	Date		Invited N	Responded n	%	Date		Invited N	Responded n	%
**W1**	19/03/2020	SDCC	479	251	52	07/04/2020	Mothers	53,968	14,075	26
		DIF	1951	1115	57		Children	53,323	13,002	24
		Total	2430	1366	56		Total	107,291	27,077	25
**W2**	26/03/2020	SDCC	251	220	88	15/04/2020	Mothers	14,079	10,414	74
		DIF	1115	862	77		Children	11,807	6,858	58
		Total	1366	1082	79		Total	25,886	17,272	67
**W3**	03/04/2020	SDCC	251	205	82	22/04/2020	Mothers	10,319	8,260	80
		DIF	1115	787	71		Children	6,422	4,241	66
		Total	1366	992	73		Total	16,741	12,501	75
**W4**	16/04/2020	SDCC	251	195	78	29/04/2020	Mothers	8,138	6,826	84
		DIF	1115	782	70		Children	3,893	2,833	73
		Total	1366	977	72		Total	12,031	9,659	80
**W5**	20/05/2020	SDCC	251	179	71	06/05/2020	Mothers	6,769	5,768	85
		DIF	1115	722	65		Children	2,760	2,193	79
		Total	1366	901	66		Total	9,529	7,961	84
**W6**	NA					13/05/2020	Mothers	5,705	4,868	85
							Children	2,143	1,724	80
							Total	7,848	6,592	84
**W7**	NA					17/06/2020	Mothers	4,848	4,427	91
							Children	1,692	1,532	91
							Total	6,540	5,959	91

SDCC= Steno Diabetes Center Copenhagen, DIF=Danish Diabetes Association

Supplemental material 2

Supplemental material 2: Distribution of age, sex, and region in the Danish population and the general population – time-series data

	Danish population aged 18–87, April 2020*		General population, time-series data	
	N	(%)	N	(%)
**Age group**				
18–29	921,178	20	227	22
30–39	688,522	15	115	11
40–49	754,721	16	166	16
50–59	800,074	17	187	18
60–69	663,751	14	165	16
70–79	567,319	12	163	16
80–87	204,792	4	23	2
**Sex**				
Men	2,283,302	50	514	49
Women	2,317,055	50	532	51
**Region**				
Capital Region (Hovedstaden)	1,460,789	32	329	31
Region Zealand (Sjælland)	663,937	14	157	15
Region of Southern Denmark (Syddanmark)	965,723	21	221	21
Central Denmark Region (Midtjylland)	1,040,981	23	233	22
North Denmark Region (Nordjylland)	468,927	10	106	10
**Total**	**4,600,357**	**100**	**1,046**	**100**

* The figures are taken from Statistics Denmark, 24 June 2020: https://statistikbanken.dk/statbank5a/selectvarval/define.asp?PLanguage=0&MainTable=FOLK1A&TabStrip=Select

Supplemental material 3: Changes in the eight crisis-specific worries

Changes in 'You will become seriously ill'

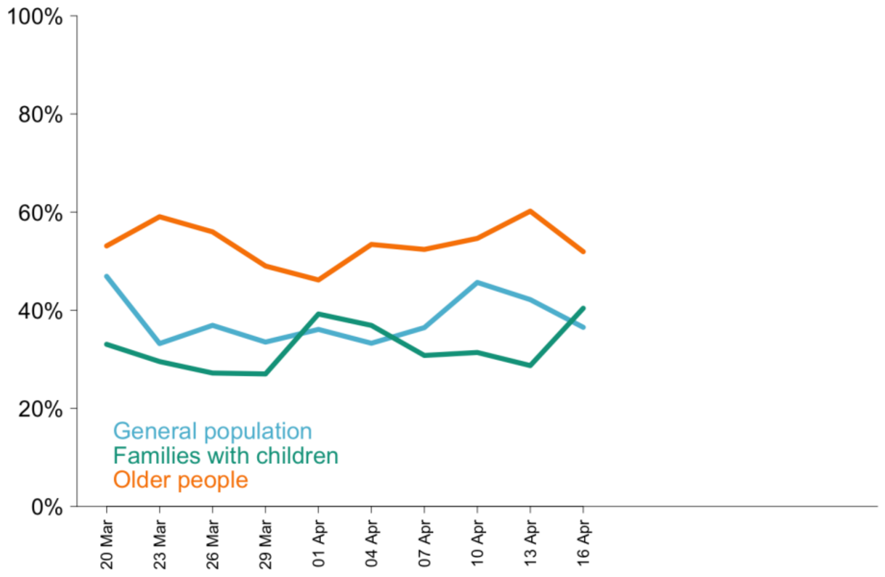

Changes in 'Someone close to you will become seriously ill'

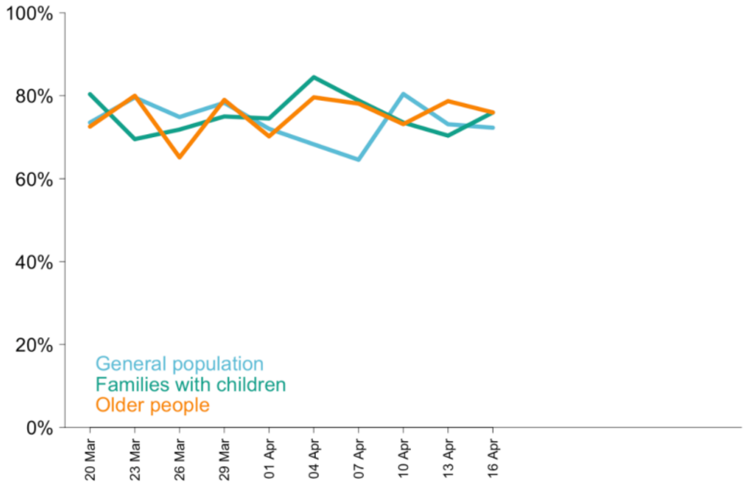

Changes in 'You or your family will experience serious financial problems'

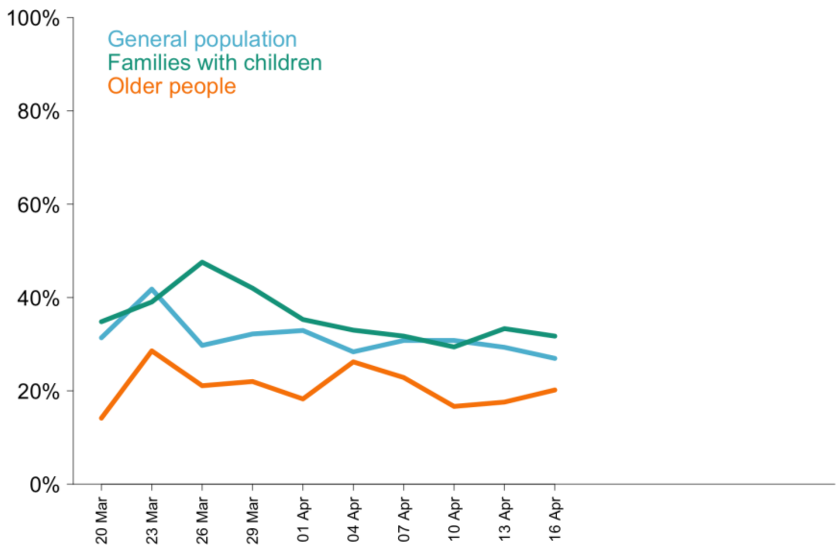

Changes in 'You will lose your job'

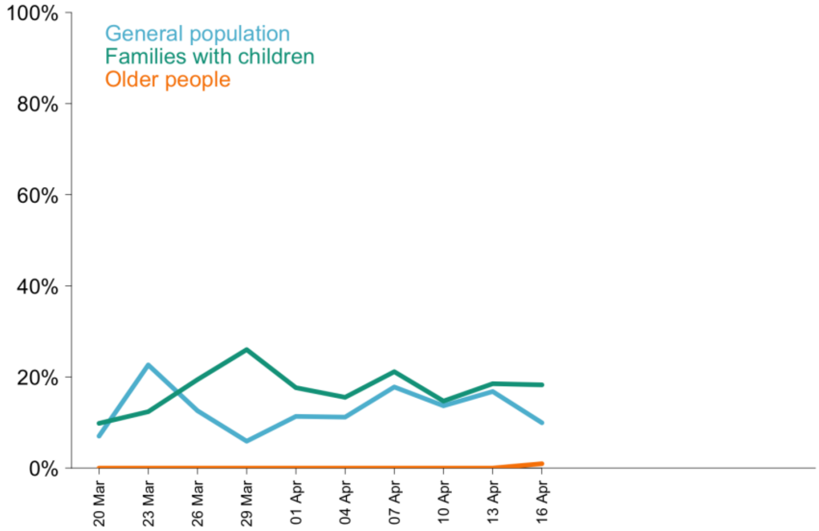

Changes in 'It will be a long time before you can resume your regular everyday life'

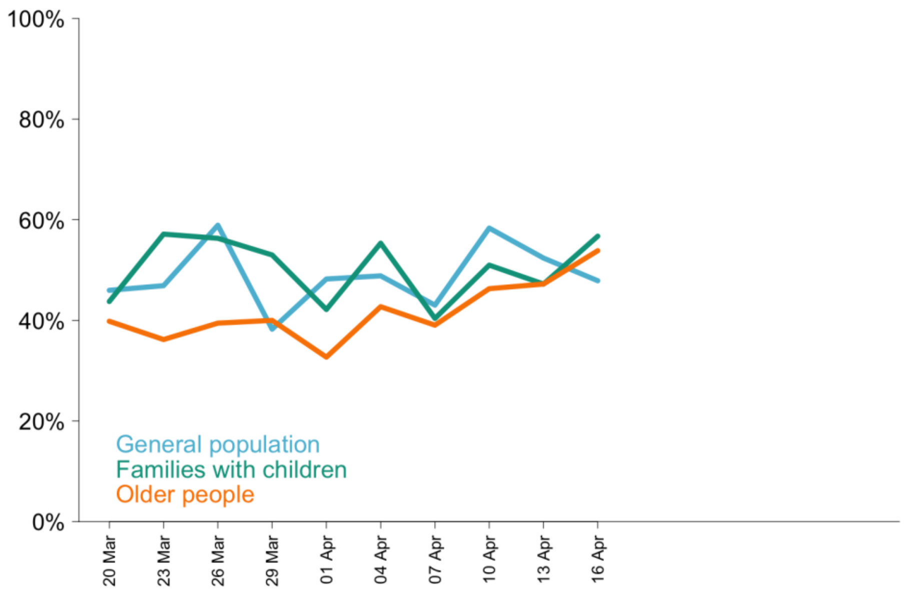

Changes in 'You will not be able to see family/friends'

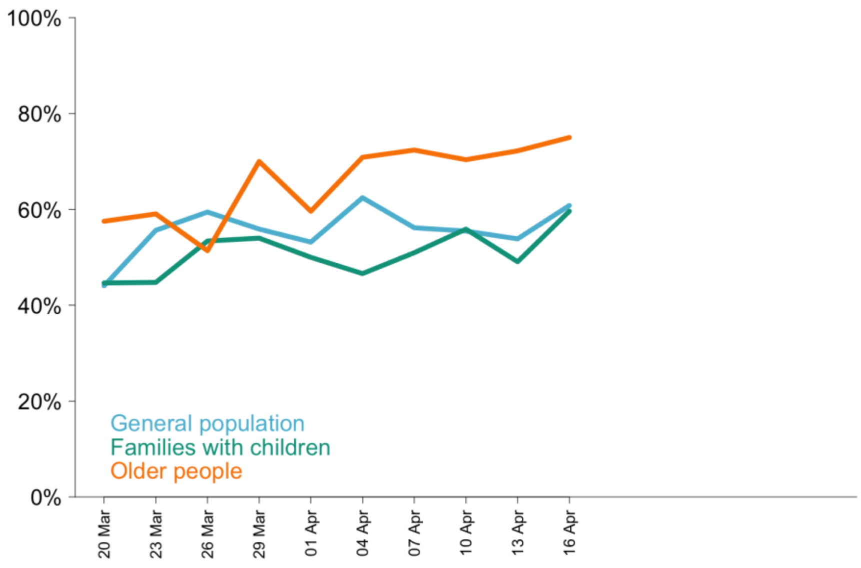

Changes in 'A national economic crisis'

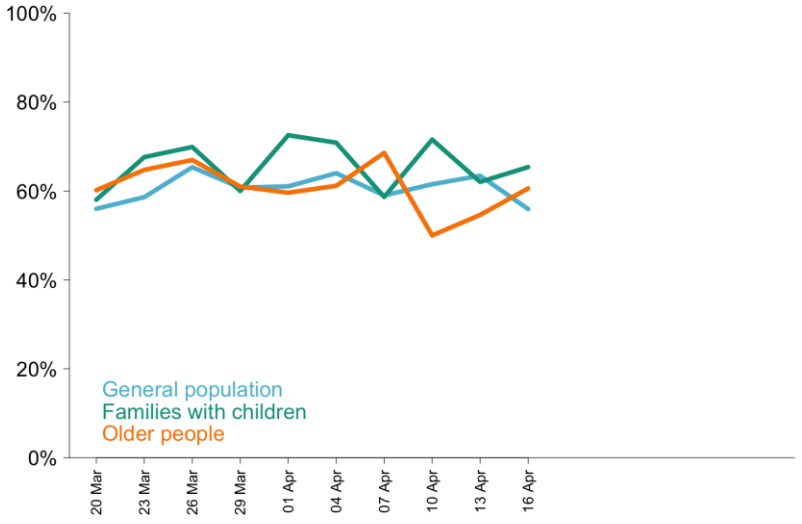

Changes in 'I am not concerned about the corona crisis'

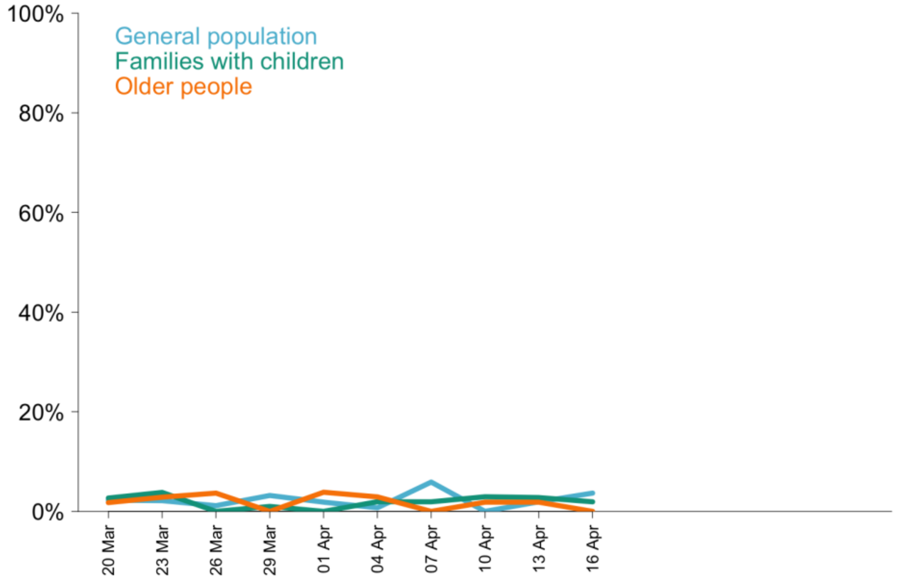

Supplement: CEP956445_Supplemental_material – Supplemental material for ‘Standing together – at a distance’: Documenting changes in mental-health indicators in Denmark during the COVID-19 pandemic [file CEP956445_Supplemental_material.pdf]
